# The Effects of City Streets on an Urban Disease Vector

**DOI:** 10.1371/journal.pcbi.1002801

**Published:** 2013-01-17

**Authors:** Corentin M. Barbu, Andrew Hong, Jennifer M. Manne, Dylan S. Small, Javier E. Quintanilla Calderón, Karthik Sethuraman, Víctor Quispe-Machaca, Jenny Ancca-Juárez, Juan G. Cornejo del Carpio, Fernando S. Málaga Chavez, César Náquira, Michael Z. Levy

**Affiliations:** 1 Center for Clinical Epidemiology & Biostatistics - Department of Biostatistics & Epidemiology, University of Pennsylvania Perelman School of Medicine, Philadelphia, Pennsylvania, United States of America; 2 Department of Statistics, The Wharton School University of Pennsylvania, Philadelphia, Pennsylvania, United States of America; 3 Department of Global Health and Population, Harvard School of Public Health, Boston, Massachusetts, United States of America; 4 Facultad de Ciencias y Filosofia, Universidad Peruana Cayetano Heredia, Lima, Peru; 5 Dirección Regional del Ministerio de Salud, Arequipa, Peru; University of Texas at Austin, United States of America

## Abstract

With increasing urbanization vector-borne diseases are quickly developing in cities, and urban control strategies are needed. If streets are shown to be barriers to disease vectors, city blocks could be used as a convenient and relevant spatial unit of study and control. Unfortunately, existing spatial analysis tools do not allow for assessment of the impact of an urban grid on the presence of disease agents. Here, we first propose a method to test for the significance of the impact of streets on vector infestation based on a decomposition of Moran's spatial autocorrelation index; and second, develop a Gaussian Field Latent Class model to finely describe the effect of streets while controlling for cofactors and imperfect detection of vectors. We apply these methods to cross-sectional data of infestation by the Chagas disease vector *Triatoma infestans* in the city of Arequipa, Peru. Our Moran's decomposition test reveals that the distribution of *T. infestans* in this urban environment is significantly constrained by streets (p<0.05). With the Gaussian Field Latent Class model we confirm that streets provide a barrier against infestation and further show that greater than 90% of the spatial component of the probability of vector presence is explained by the correlation among houses within city blocks. The city block is thus likely to be an appropriate spatial unit to describe and control *T. infestans* in an urban context. Characteristics of the urban grid can influence the spatial dynamics of vector borne disease and should be considered when designing public health policies.

## Author Summary

Chagas disease is a major parasitic disease in Latin America. It is transmitted by *Triatoma infestans* an insect common in Arequipa, the second largest city in Peru. We propose a method to demonstrate that streets strongly affect the spatial distribution of infestation by this insect in Arequipa. The effect of streets may be due to several external factors: 1) houses on different sides of a street may not be equally welcoming to the insects due to the presence of certain materials or animals, 2) people inspecting houses on the two sides of a street may not be equally efficient, and, 3) insects may disperse to neighboring houses but rarely reach houses across a street. We take these aspects into account in a second analysis and confirm that streets are important barriers to these insects. Our finding should allow for improvements in the control of insects that transmit Chagas disease in cities. More generally, our methods can be applied to other pests and disease vectors to better understand and control epidemics in cities.

## Introduction

In the context of increasing urbanization worldwide [1]–[4], vector-borne diseases, a significant burden to human and animal populations [5], are quickly emerging in cities [6] and require the adaptation of control strategies to densely populated and highly interconnected environments. Notable examples include Dengue [7]–[9], Malaria [10]–[12] and Chagas disease [13]–[15].

Prevention of vector-borne diseases relies heavily on vector control [8], [16], [17]. Given the substantial resources needed to control vector populations on the city scale, well-managed control strategies based on understanding of vector spatial dynamics can potentially increase cost efficiency [18]–[20]. A central feature of cities is the grid of streets which fractures the environment. Such disjoint landscapes can affect patterns of occurrence and transmission of diseases, both communicable and non-communicable. Assessing the impact of the urban grid on the spatial distribution of diseases could lead to more effective design of surveillance and control programs in cities.

Arequipa, Peru, a city of nearly 1 million inhabitants, is currently experiencing an epidemic of infestation by *Triatoma infestans* [21], [22], the principal vector of *Trypanosoma cruzi* [23], the etiological agent of Chagas disease [24]. The spread of *T. infestans* in Arequipa is accompanied by micro-epidemics of *T. cruzi* transmission to humans [25], [26]. Control of the vector in the city through insecticide application in households is challenging [20], [27]. Previous work on *T. infestans* and other Chagas disease vectors have used non-spatial [28], [29] and spatial modeling techniques [19], [30]–[33] to characterize vector population dynamics and propose improvements in control strategies. However, these studies have not considered the impact of an urban grid on vector populations.

The impact of known boundaries such as roads or rivers on epidemics or population dynamics has occasionally been assessed using spatio-temporal modeling to describe spatio-temporal presence-absence data [34]. Using only spatial data, kriging approaches integrated in well known GIS softwares may take into account the presence of known landscape features as impenetrable barriers [35] but do not assess the resistance of these barriers. In landscape genetics, the quantification of the effect of barriers is a central aim of a large and growing field [36]–[38]. These approaches benefit from the complex information present in DNA to infer the impact of barriers. Some of their results, however, depend on the assumption of migration-drift equilibrium which is typically violated in epidemics and highly dynamic human influenced landscapes [39]. In social sciences, disparities among spatially well circumscribed census tracts are commonly quantified using indices of segregation [40], but the borders between such tracts are not usually considered as barriers themselves [41].

Here we propose to quantify the impact of known boundaries by measuring their effect on spatial autocorrelation in presence-absence data. Variations of the autocorrelation over distance have been measured and presented in autocorrelograms [42], [43]. Another approach has been to parameterize kernels describing these variations, notably to produce Bayesian disease risk maps [44]–[46]. We extend both of these approaches to assess the impact of known barriers such as streets on the spatial distribution of binary data, in our case the presence of *T. infestans* in households of the city of Arequipa. First, we provide a global assessment of the effect of streets on vector infestation using a decomposition of the commonly used Moran's I statistic and corresponding autocorrelograms [47], [48]. Second, we capture the effect of streets on a finer scale and control for cofactors and imperfect detection of vectors by designing a Gaussian Field Latent Class model. Taking into account streets, the kernel of this model describes the spatial correlation through precision matrices [49], [50] in the framework of a spatial Bayesian Generalized Linear Model [51]. Finally, we discuss how surveillance and control of Chagas disease in cities can be better informed by taking into account the impact of streets on infestation.

## Materials and Methods

### Entomological Data Collection and Mapping

We conducted our study in Paucarpata, the largest district in the city of Arequipa, Peru. The Ministry of Health of Arequipa applied insecticide to 13,917 households in Paucarpata between November 2006 and April 2009. During the insecticide application campaign, household-level data on the presence or absence of *T. infestans* and relevant risk factors for vector infestation were collected. Risk factors included a description of construction materials in each house and the presence of guinea pigs, dogs, and other domestic animals.

We mapped the position of all households and the delimitation of city blocks in the district comparing satellite imagery in Google Earth™ [52] to field maps drawn by the personnel of the Ministry of Health. Households were then snapped to their city block according to their respective coordinates.

### Statistical Analysis

#### Application of Moran's I to an urban grid

We first assessed the impact of streets on the global spatial autocorrelation of vector infestation as measured by the Moran index (I) [47]. This index reads:
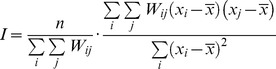
where 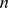 corresponds to the number of households, 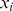 indicates the presence (1) or absence (0) of insects in household 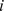; 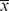 is the mean of the observations over all households, and 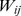 represents the weights describing the spatial relationship between households 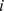 and 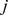. 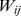 is set to 1 if the distance between point 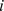 and point 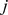 falls within a given range; otherwise, it is set to 0. We calculated autocorrelograms [43], [53] for the occurrence of *T. infestans* in Paucarpata for successive 15m-wide distance ranges.

To determine if streets affect the spatial autocorrelation of infestation, we decomposed the autocorrelation into a within city-block component and an across city-blocks component. We then calculated separate autocorrelograms for pairs of households on the same city block (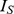) and for pairs of households on different city blocks (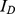).

We then assessed the significance of the difference 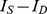 using the following random labeling permutation test [54]. For a given distance range, each household has 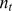 total neighbors, among which 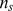 are on the same city block. For each permutation we randomly assigned 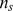 of the 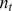 neighbors as occupying the same city block as the index house, and the remainder of neighbors as occupying a different city block. We then calculated the corresponding 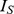 and 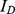. We repeated this process for 1000 permutations, creating a histogram of the values of 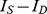. We determined the p-value of our observed value of 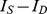 by referencing this histogram. We applied this decomposition of Moran's I to all the households participating in the vector control campaign.

The decomposition of Moran's Index offers a fast, simple way to obtain an estimate of how streets impact the autocorrelation of observations. However, several factors could confound or obscure this estimate. First, well-known risk factors for *T. infestans* presence, such as construction materials or presence of domestic animals [27], may be more common on some city blocks than others. Such an aggregation of cofactors could contribute to the structure of vector populations. Multivariate methods are needed to tease apart the effect of such cofactors from that of city streets. Second, due to the vast areas surveyed, multiple inspectors are employed to search houses for vectors. These inspectors may vary in their ability to detect insects. If some city blocks are examined by more sensitive inspectors and others by less sensitive ones, the observed spatial distribution of infestation may be structured, even if the full, unobserved distribution of infestation is not.

Beyond these two considerations, there is a third, less obvious limitation to the Moran's I. As a pair-wise statistic, Moran's I, as well as its derivatives described here, measures indirect and direct correlation together: measured correlation could result either from a direct correlation between households or an indirect correlation mediated by the in-between households that are strongly correlated on a small distance scale. The effect of streets can be important simply because streets create a gap in a chain of small distance scale autocorrelations between households (hereafter the “gap effect”). In contrast, streets may, above and beyond the gap effect, serve as a barrier to vector migration (hereafter the “barrier effect”). A spatial field-based measure of autocorrelation accounts for the autocorrelation of neighbors at all distances simultaneously. Such an approach can then detect a barrier effect linked to the presence of streets and not only to the uneven distribution of households induced by streets.

#### Application of a Gaussian field model to an urban grid

**Figure 1:**
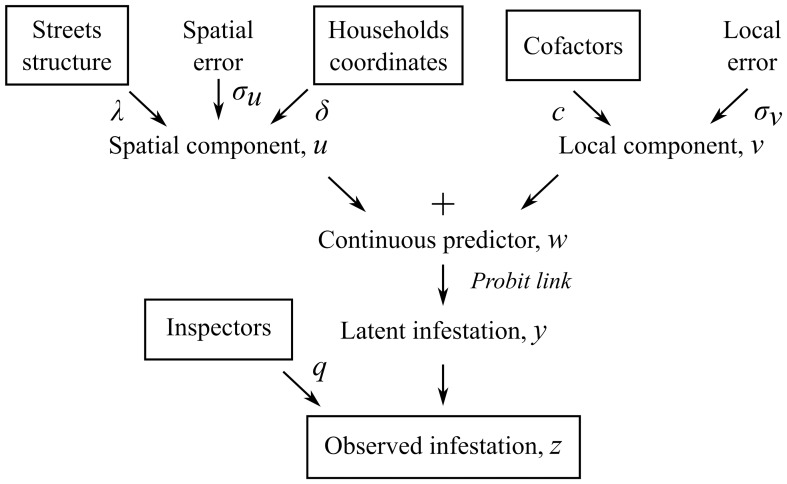
General structure of the Gaussian Field Latent Class model. Working backward, we consider the infestation data 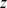 to be the result of a latent infestation status 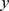, observed by imperfect inspectors of sensitivity 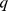. The true infestation 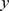 is a binary manifestation of an underlying continuous infestation predictor 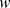. Cofactors and a local error term, 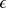, form the local component. The spatial component 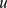 is modeled as a Gaussian field. The fit parameters, 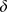 and 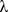, respectively tune how distances between neighbors and the streets define the spatial dependency between households in the spatial component.

We built a Bayesian generalized linear model describing household infestation status as a discrete manifestation of a continuous predictor of infestation. The predictor of infestation includes a spatial field component [51], accounting for the street network, and a non-spatial component, integrating local cofactors. Additionally, household infestation status is considered as a latent class [55], [56] to account for the imperfect sensitivity of the inspectors surveying the households. We refer to our model, shown in Fig. 1, as a Gaussian Field Latent Class model. Hereafter upper case characters indicate matrices, lower case characters vectors, and Greek letters scalars.

Spatial component. The spatial component *u* of the infestation predictor 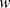 is an auto-regressive Gaussian Markov random field described through its precision matrix [49], [50]: for any household *i*, the mean of the spatial component, 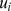, is a weighted mean of the spatial components of its neighbors, weighted by the distance to them. Normal variations around the spatial mean are allowed, their variance increasing with the isolation of the household (mathematical details on the Gaussian Markov random field are provided in Section 1 in Text S1).

We introduce the effect of streets in a similar way as in the decomposition of the Moran's I – by distinguishing between neighbors within a city block and proximate households separated by streets. The spatial weight 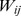 between the households 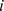 and 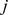 is:
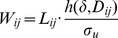
where 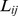 takes the value 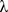 if 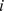 and 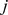 are on different city blocks and the value 1 if they are on the same block; 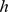 is a spatial kernel of shape factor 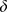 applied to the distance 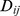 between the households and 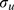 is a scale parameter for the spatial error.

We consider four one-parameter kernels describing a wide range of shapes (Table 1). For computational reasons, when the distance 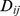 is above a distance threshold 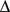 (set at 100 m) the households are considered to have no direct influence on each other and thus their weights are set to 0. A sensitivity analysis (Section 2 in Text S2) shows that the choice of 100 m as a threshold provides in our case a robust estimate of the parameters of interest.

**Table 1:** Spatial kernels and corresponding fitted parameters.

Name	Equation, 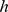	Shape	DIC	Shape factor^a^, 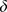	Streets factor, 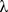	Same Block Index^b^
Exponential	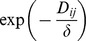	Sharp top, thin tail	2526	9.00 (7.04–11.8)	0.30*** (0.12–0.61)	94.0% (89.8–96.9)
Gaussian	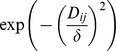	Flat top, thin tail	2553	17.3 (14.4–21.2)	0.52  (0.16–1.24)	93.7% (88.1–97.3)
Cauchy	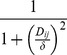	Flat top, fat tail	2553	8.25 (5.30–13.0)	0.08*** (0.04–0.14)	94.7% (91.4–97.3)
Geometric	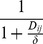	Sharp top, fat tail	2609	7.64 (2.08–26.3)	0.03*** (0.01–0.05)	95.1% (91.6–98.2)

a The shape factor 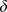 is indicated in meters.

b Same Block Index: Percent of the spatial component of infestation explained by same city block neighbors (see Section 2 in Text S1). In parentheses are the 95% Credible Intervals (CrI) according to the MCMC sampling. The probability of having no barrier effect of streets is indicated with the values of 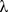: 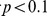;

***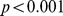.

To assess the relevance of the city-block as a spatial unit of infestation we calculate the “Same Block Index” which we define as the mean percentage of the spatial component of infestation explained by neighbors on the same city block (Section 2 in Text S1).

Local component. We include in a local component 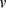 the effects of known cofactors and a local error term 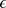:
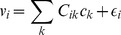
with 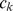 the risk coefficient for the presence of the cofactor 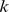, 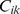 the indicator of presence of the cofactor and 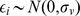 with 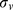 the scaling parameter of the local error (see Section 3 in Text S1).

Link function. We relate our outcome data, the observed infestation, 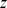, to the continuous infestation predictor 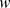, in two steps. A probit function links the infestation predictor 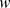 to the latent infestation status 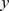: 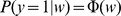 where 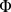 is the Cumulative Distribution Function of the standard normal distribution. The latent infestation is then imperfectly revealed by the inspection: an infested household is observed as infested by an inspector 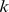 with a probability 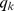: the sensitivity of the inspector.

When infestation data are not available (non-inspected houses), the sensitivity is set to 0 (see Section 4 in Text S1 for more details on the implementation and Section 2 in Text S2 for an analysis of the sensitivity of the results to this modeling choice).

**Figure 2:**
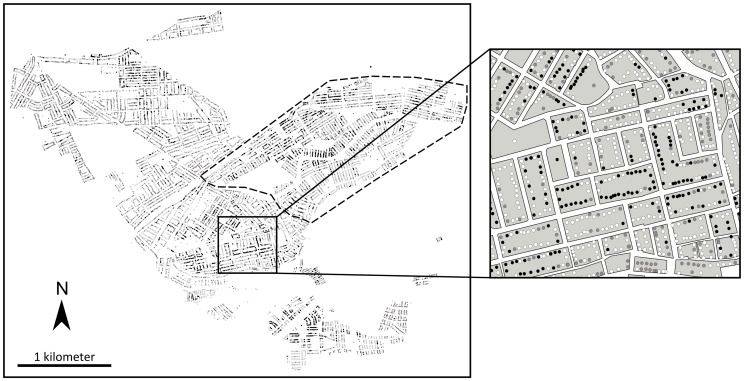
Spatial distribution of *Triatoma infestans* presence in households of Paucarpata, Arequipa, Peru. Map of the study area. Black indicates infested households, white non-infested households, and grey non-inspected households. The area encircled by dashes was used to fit the Gaussian Field Latent Class model; the remaining area was used as a validation dataset. The close-up shows the urban grid underneath and the aggregation of vectors within city blocks.

Fitting and validation. We fit the Gaussian Field Latent Class model on a fraction of the map consisting of all of the households inspected between September and December 2007 (Fig. 2). We used the remaining households as a validation dataset. For all priors, we use flat or weakly informative priors [57]. Further mathematical details on the implementation of the sampling are given in Section 5 in Text S1.

We used the validation dataset to determine how well our parameterized model predicted the presence of vectors in unobserved households. To do so, we randomly selected 5% of the houses in the validation set and removed them. We set the sensitivity of inspectors and the spatial parameters to their estimated means, remove the cofactors from the model and refit the spatial component, predicting the observation of infestation in the withheld households. We repeated the process 20 times, without replacement, so that all houses had been selected for prediction exactly once. We then evaluated the predictions using the McFadden index [58].

As a second check, we verified that the Gaussian Field Latent Class model properly reproduced the global autocorrelation of the observed infestation by generating 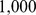 vector infestation maps across the validation dataset and repeating the Moran's I analysis on each.

All analyses were performed in R [59]; the code is available in Data S1 and updated versions are available at https://github.com/cbarbu/spatcontrol.

## Results

During the vector control campaign in Paucarpata, the Ministry of Health sprayed 9,654 houses, among which 1,791 (18.5%) were infested with *T. infestans* (Fig. 2). Data was unavailable from an additional 4,263 (30%) households, most of which chose not to participate in the spray campaign.

### Importance of streets as assessed by the decomposition of Moran's index

**Figure 3:**
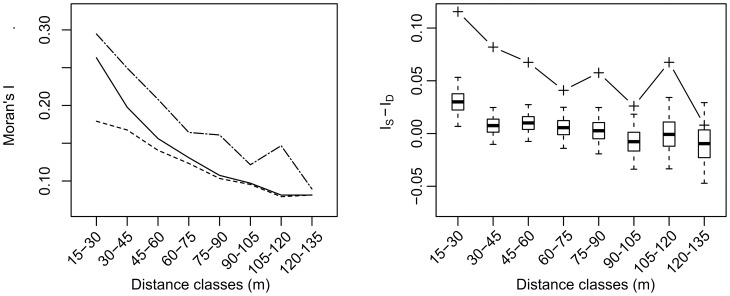
Spatial autocorrelation of *Triatoma infestans* presence in Paucarpata, Arequipa, Peru and the effects of streets. Left: autocorrelation of the infestation status as a function of the distance. Solid line: Global Moran's index. Dot-Dashed line: Moran's Index for within blocks household pairs. Dashed line: Moran's Index for household pairs across streets. All Moran's I values are significantly different from the expected value under hypothesis of no spatial autocorrelation (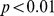). Right: significance of the difference between the correlation within city blocks and the correlation across streets. Box plots indicate the expected values under the null hypothesis using a permutation test. The boxes indicate the 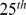, 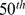 and 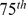 quantiles, and the whiskers depict the 95% CrI.

For all distance classes up to 120 m, the autocorrelation among houses within the same city block was significantly greater than that among houses separated by streets (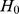: spatial autocorrelation independent of streets, 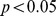) (Fig. 3). For the distance class above 120 m, the difference was not significant, probably due to decreased sample size of same city block neighbors. Interestingly, the autocorrelation across a street is consistently similar to the autocorrelation within a same block 30–45 m further.

Notably the expected difference 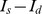 under the null hypothesis is positive at short distances. This unintuitive result is due to the non-negligible width of the rings we used to bin our data: at short distances, houses across streets are further from each other than those on the same block, hence there is a slightly positive expectation for the difference in Moran's I. Our permutation test reproduces this effect and thereby controls for it in determining the significance of the impact of streets.

### Importance of the barrier effect of streets as assessed by the Gaussian field model

Controlling for the spatial distribution of cofactors and inspectors, we estimated the barrier effect of streets on infestation to induce a two to thirty fold decrease (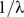) in the spatial weight between households for a given distance, depending on the chosen kernel (Table 1). The “Same Block Index”, quantifying the relevance of the city-block as a spatial unit of infestation, exceeded 90% and our estimate was extremely robust across all four kernels considered (Table 1).

### Cofactors

As a part of the fitting process of the Gaussian Field Latent Class model, we assessed the effect of cofactors on the presence of vector infestation. We found that the presence of guinea pigs and the presence of dogs were significant risk factors for vector infestation. Conversely, we found that the presence of plastered walls inside of the house was strongly and significantly protective against infestation. The degree of the effect of these cofactors varied across the four kernels considered (detailed results in Table S1).

Interestingly, for all four kernels, the standard deviation of the continuous infestation predictor induced by the joint effect of all the cofactors and the random effect (0.44–0.54) was threefold less than the standard deviation of the estimated spatial component across households (1.64–1.93).

### Inspector sensitivity

We also assessed the quality of inspectors in terms of their sensitivity—the probability that an inspector detects vectors in households that are indeed infested. The mean inspector sensitivity was 70%, with extremes at 41 and 90% (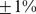 depending on the kernel). The relative ranking of inspectors by their sensitivity was largely preserved across kernels, and the estimates of inspector sensitivities did not vary greatly (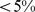) between models with alternative kernels.

### Model validation

**Figure 4:**
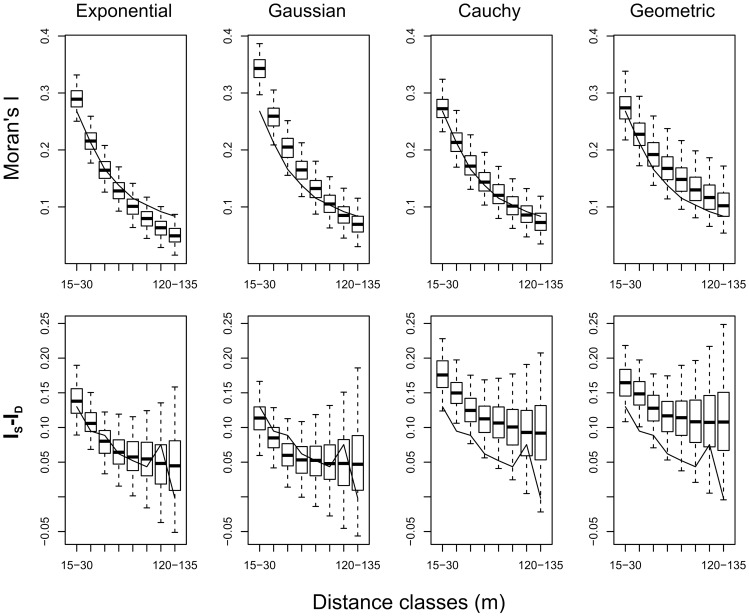
Spatial autocorrelation of data simulated with the Gaussian Field Latent Class model of *Triatoma infestans* distribution. The autocorrelation of infestation in the generated data is compared to the autocorrelation in observed data. Infestation data were generated on the validation map using the estimated parameters for each of the kernels: exponential (first column), Cauchy (second column), Gaussian (third column), and geometric (fourth column). We calculated the standard Moran's I (first row) and the difference 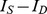 between within block and across street autocorrelation (second row) as a function of distance. The solid line indicates the values for the observed data. Box plots indicate the values obtained from generated data. The boxes indicate the 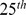, 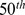 and 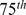 quantiles, and the whiskers depict the 95% CrI.

The Gaussian Field Latent Class model allowed us to make generally accurate predictions in hold-out households across the four kernels (McFadden index [58] of 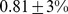 depending on the kernel). The model also reproduced the patterns observed with the Moran's I analysis across all four kernels, both in terms of classical Moran's I and of decomposed Moran's I (Fig. 4). Differences can nevertheless be observed between the kernels. In particular, the exponential kernel closely reproduced the global autocorrelation up to 75 m and the impact of streets on the spatial autocorrelation (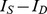) at all distances. The DIC values [60] obtained with the respective kernels (Table 1) also indicate particularly good performance of the exponential kernel.

## Discussion

We observed a significant effect of streets on the spatial pattern of Chagas disease vectors in Arequipa, Peru, and show that greater than 90% of the spatial component of infestation is determined by neighbors on the same city block. In addition, the difference of autocorrelation in the same block and between blocks indicate that the crossing of streets is grossly equivalent to an added distance of 30–45 m in terms of spatial autocorrelation. The limiting effect of streets was consistent across two methodological approaches: a pair-wise analysis (decomposed Moran's I) and a field based model (Gaussian Field Latent Class). The latter approach accounted for known cofactors and imperfect detection, further confirming that streets constitute an important barrier to aggregation of triatomine infestation above and beyond the uneven spatial distribution of urban households.

The underlying cause of the barrier effect of streets on *T. infestans* remains unclear. As we control for the spatial distribution of known cofactors, and the varying sensitivity of different inspectors surveying houses, the observed autocorrelation and its perturbation by streets are likely to be related to the movements of the insects [34], [61]. We have previously shown that the majority of *T. infestans* dispersal is due to early-stage nymphs [22]. These insects, the size of a small ant, may simply be unable to cross streets. In addition, it should be noted that the façades of houses are usually plastered, representing a barrier to dispersion of *T. infestans*, as for other insect species [62]. In contrast, the walls in the back of houses are typically not plastered, loosely stacked stones or bricks that provide hospitable habitats for vectors, and may facilitate insect movement within the block.

Several authors have commented on the need to assess the role of landscape heterogeneity in the context of epidemiological [34], [63] and ecological studies [38]. Previous work evaluating barriers to animal dispersion or disease propagation has focused on a small number of potential barriers using spatio-temporal data [64]–[66], observational and mark/recapture methods [67]–[69], or population genetics [38]. These approaches have been used to characterize the impact of roads on insect populations in rural settings and the connectivity of vertebrate populations in urban environments. Specifically, roads in open fields have been implicated as barriers for a handful of insects, including ground beetles [70]–[72], carabid beetles [67], [73], bumble bees [74], and dragonflies [75]. In urban settings, it has been shown that streets act as a barrier to hedgehog movement [68] and structure rat populations by city block [76]. Our study both extends existing approaches to these questions by providing a methodology to assess the importance of streets in the context of multi-variate models and offers evidence of the strong effect of streets on *T. infestans* populations.

We have shown previously that the presence of guinea pigs, the presence of dogs and the presence of other animals are risk factors for triatomine infestation and that fully cemented plaster walls are protective [27]. These findings held true in our current analysis, but the influence of cofactors was small compared to the spatial component of our model. Our previous studies were conducted in a peri-urban area where *T. infestans* populations were established, and exhibited no spatial clustering [27]. In the current study site, which is more urban, vector populations exhibit strong spatial clustering, suggesting that they may be expanding [61]. When populations are in a continuous dynamic of dispersal or redispersal, the effect of heterogeneous habitats is often weak compared with that of the spatial dynamics of colonization [77]. In Paucarpata, we believe vector dynamics trumped the effects of traditional cofactors, which would be more predictive of infestation in a stable system.

There are several limitations to our study. The effect of streets detected in our approach could be confounded by unmeasured cofactors strongly clustered within blocks. Two reasons nevertheless limit the probability of such a scenario. First our Gaussian Field Latent Class model explicitly accounts for the main known cofactors of infestation by Chagas vectors as identified previously [27], [52], [78], [79]. Second, taking into account these known cofactors has a limited influence on the estimated influence of streets (Section 3 in Text S2).

We were only able to obtain binary data on the presence or absence of vectors; data on vector densities could provide more information with which to assess the effect of streets. While our analysis is tailored to binary observations, it could be extended to consider discrete measurements. Our Gaussian Field Latent Class model can be applied to a wide variety of datasets without adaptation of the priors; however, care should be taken to correctly choose the order of magnitude when assigning a prior on the shape factor of the spatial autocorrelation kernel. The use of 100m as a threshold distance beyond which correlation is assumed to be null is a simplification needed to lessen computation time; we assessed the effect of this simplification and determined that our findings were not affected by it.

The flat prior used here for inspector sensitivity may shrink the posterior towards the mean of the prior, 50%. The true sensitivity is then likely greater than the estimate provided here, 70%. However, the strong estimated effect of streets is robust to variations of the prior (Section 4 in Text S2).

Further extension of the model would be necessary to determine whether wider streets pose a greater barrier to insects than narrower ones. Interestingly, if, as we hypothesize, the barrier effect is mainly due to the asymmetry in housing materials in the front and back of houses, broader streets may not pose a greater barrier to insects. Finally, further work is needed to assess if the impact of streets is affected by the seasonality of *T. infestans* dispersion. This would provide much needed biological insight given the importance of seasonality in triatomine dispersion [29], [33].

Our findings have implications for adapting control strategies to disease transmission dynamics. First, city blocks have been used as a practical unit of study previously [80]–[82], and here we show that they are a relevant spatial unit for the study of urban Chagas disease. Given the high cost of insecticide application, it may be much more efficient to develop targeted control strategies that are appropriate for the urban geography – taking greatest advantage of the barrier effect of streets. More specifically, current practices in such localized interventions are based on ring treatment that ignores the impact of streets. Our results suggest that control efforts may be more effective if they are expanded further within the same city-block (30–45m), before crossing a street: more of an “oval” treatment strategy, giving preference to houses on the same block when resources are limited. Second, as city blocks seem adequate for describing infestation and thus exposure of inhabitants to disease agents, they may also be valuable in modeling parasite transmission [83] and targeting screening for infection [21], [84]. Third, over the long term, it is expected that resistance to pyrethroid insecticides will be observed in urban settings as it has already been in rural areas [85]–[88]. The fragmentation of the vector population by streets may then affect the propagation of resistance alleles [89]. Finally, we expect the distribution of streets to affect the dynamics of the vector spread both in terms of speed, as different localities have different densities of streets, and direction, as city-blocks are usually twice as long as they are wide across a neighborhood.

The difficulties presented in controlling Chagas vectors in cities are similar to those of other urban disease vectors and pests such as the mosquito vectors of Dengue (*Aedes aegypti*) and bed bugs (*Cimex lectularius*). The effect of streets and other aspects of the urban environment should be considered when designing control or elimination campaigns against these organisms.

## Supporting Information

Data S1 **Annotated code in R with data.** Two datasets are given. One corresponds to the original, de-identified data (realData.R) where spatial relationships are given through the distance matrix between points. The second dataset (JitteredDataPaucarpata.csv) corresponds to a generated dataset having the same spatial characteristics as the original dataset. The given examples with this second dataset (example_structuredMI.R and example_fit_GMRF.R) provide a model for application of these methods to new datasets. Please see the README file for details. Updated code can be accessed through github: https://github.com/cbarbu/spatcontrol.
(ZIP)

Table S1 **Detailed parameters estimates.** Estimates for each spatial kernel of all fitted parameters with their 95% credible intervals.
(PNG)

Text S1 **Details about the mathematical model.** Detailed description of the Latent Class Gaussian Field implementation (model and sampling).
(PDF)

Text S2
**Sensitivity analyses.** We further investigate the sensitivity of the Latent Class Gaussian Field model to the distance threshold 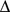, the handling of missing data, the included cofactors, and the inspector sensitivity prior.(PDF)
