# Supplementary figures and images for: The Effects of City Streets on an Urban Disease Vector
Source: PLoS Comput Biol. 2013 Jan 17;9(1):e1002801. doi: 10.1371/journal.pcbi.1002801 (PMC3547802; doi:10.1371/journal.pcbi.1002801)

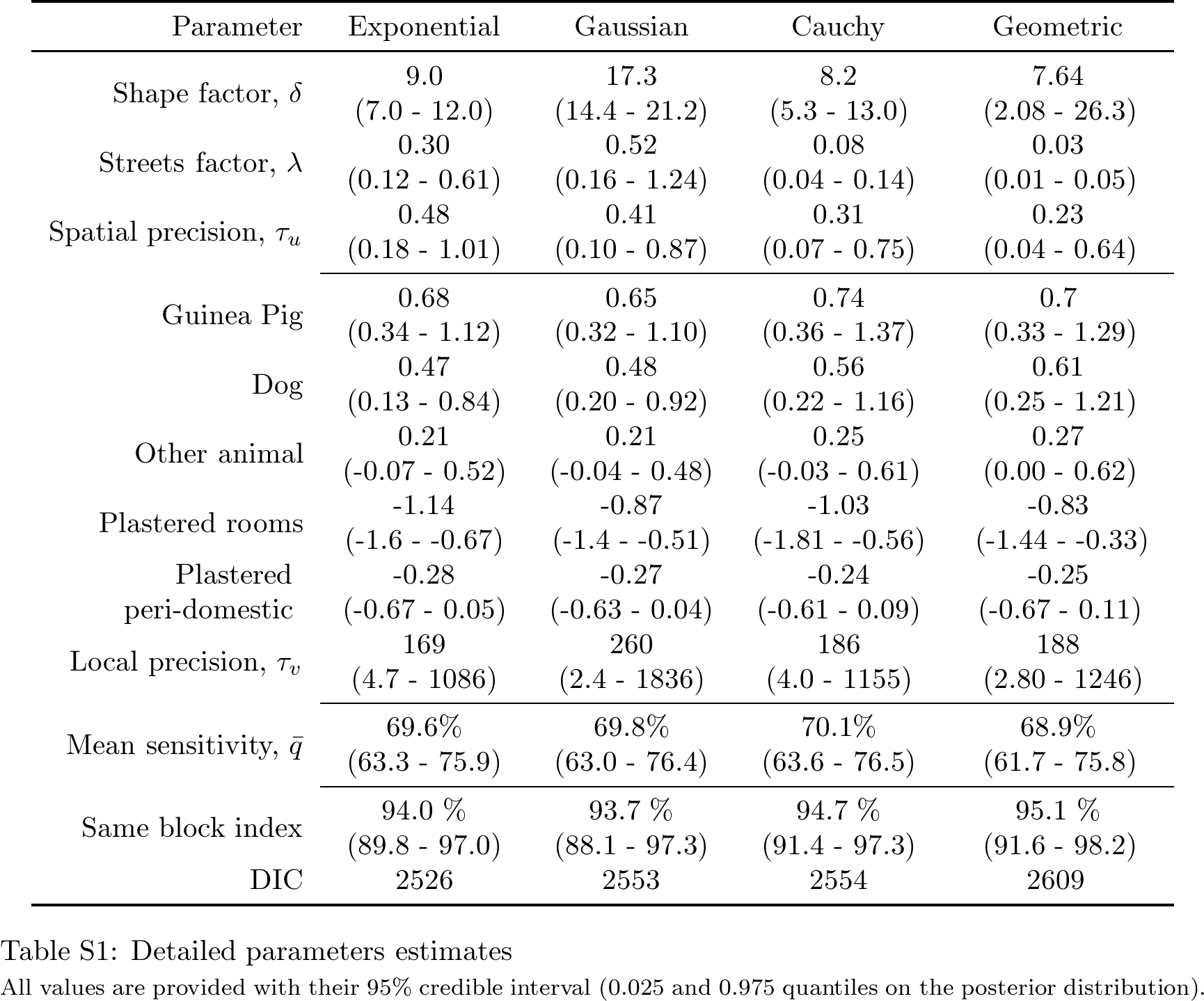

Supplement: Table S1 — Detailed parameters estimates. Estimates for each spatial kernel of all fitted parameters with their 95% credible intervals. (PNG) [file pcbi.1002801.s002.png]
