# Supplementary material for: The Effects of City Streets on an Urban Disease Vector
Source: PLoS Comput Biol. 2013 Jan 17;9(1):e1002801. doi: 10.1371/journal.pcbi.1002801 (PMC3547802; doi:10.1371/journal.pcbi.1002801)
Supplement: Text S1 — Details about the mathematical model. Detailed description of the Latent Class Gaussian Field implementation (model and sampling). (PDF) [file pcbi.1002801.s003.pdf]

Supplementary Materials Text S1:
Effect of City Streets on an Urban Vector
Detailed Description of the
Gaussian Field Latent Class Implementation

C.M.Barbu et al.

Contents

1 Spatial component **1**

2 The city-block as a spatial unit **3**

3 Local component of the infestation predictor **4**

4 Observation by imperfect inspectors **4**

5 Sampling of the Gaussian Field Latent Class model **4**

The Gaussian Field Latent Class model we developed links the observed infestation z to the city street structure, the inter-household distances, the presence of known cofactors of households infestation, and the inspectors surveying the households (Fig. S1.1).

1 Spatial component

The spatial component is based on an adaptation of the auto-regressive Gaussian Markov random Field model [1] to allow the estimation of the influence of streets on the spatial auto-correlation structure of infestation. In a Gaussian Field model the spatial component u of the continuous infestation predictor w follows a multivariate normal distribution with a centered prior of precision (inverse covariance) matrix Q (also called concentration matrix [2]). Each coefficient Q_{ij} corresponds, in our case, to the opposite of the weight W_{ij} between a household i and a household j as defined in the main text. The auto-regressive model additionally assumes that $Q_{ii} = \sum_{j \neq i} Q_{ij}$. From this choice, a particularly

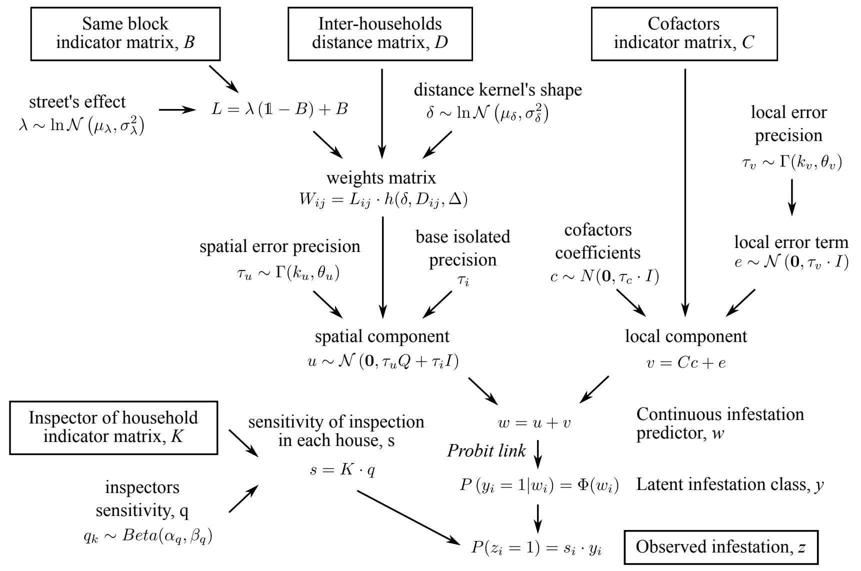

Figure S1.1: **Full description of the Gaussian Field Latent Class model.** We present here the information integrated into the model (shown in boxes) as well as the priors on each parameter. $\mathcal{N}$ stands for the multivariate normal distribution, parameterized with its mean vector and its precision matrix. Γ stands for the gamma distribution with, in order, its shape and scale parameters. $\ln\mathcal{N}$ stands for the log-normal distribution with, in order, its log-scale and shape parameters. Φ is the cumulative distribution function of the standard normal distribution. For a number of houses n, $\mathbf{0}$ is the null vector of size n, I is the identity matrix, and $\mathbb{1}$ is the matrix of ones, both of size $n \times n$. Parameters are detailed in the text.

interpretable marginal distribution follows:

$$\forall i \;\; u_i | \mathbf{u}_{-i} \sim \mathcal{N}\left(\frac{\sum\limits_{j \neq i} Q_{ij} u_j}{\sum\limits_{j \neq i} Q_{ij}}, \frac{1}{\sum\limits_{j \neq i} Q_{ij}}\right) \tag{1}$$

The spatial component, u_i, for a household i given the value of the spatial components in all other households $\mathbf{u}_{-i}$ follows a normal distribution. Its mean is a weighted mean of the spatial component in its neighbors. The variance around this mean being inversely proportional to the sum of the weights implies that the determination of the spatial component by neighboring households is relaxed for isolated houses.

The auto-regressive condition $Q_{ii} = \sum\limits_{j \neq i} Q_{ij}$ implies that the precision matrix Q is not invertible. It also implies the spatial precision for isolated households

$\sum_{j \neq i} Q_{ij} = 0$: the marginal variance for these isolated households is infinite. To avoid these problems we add an epsilon $\tau_i = 0.01$ to the diagonal. The prior on the spatial variance, particularly important for isolated households, is then 100 and no more infinite, and a determinant can subsequently be computed for Q. This second aspect allows computation of the likelihood of Q given u:

$$\pi(Q|u) = \frac{|Q|^{-1/2}}{(2\pi)^{1/2}} exp\left(-\frac{1}{2}u^T Qu\right)$$

with $\begin{cases} \forall i \neq j & Q_{ij} = -W_{ij} \\ \forall i & Q_{ii} = \tau_i + \sum_{j \neq i} W_{ij} \end{cases}$ and as defined in the main text: $W_{ij} = L_{ij} \cdot \frac{h(\delta, D_{ij})}{\sigma_u}$ where L_{ij} takes the value λ if i and j are on different city blocks and the value 1 if they are on the same block; h is a spatial kernel of characteristic distance δ applied to the distance D_{ij} between the neighbors and σ_u is a scale parameter for the spatial error of prior $\sigma_u \sim \Gamma(k_u, \theta_u)$ with $k_u = 0.001$ and $\theta_u = 1000$ corresponding to a weakly informative prior on the scale parameter around 1.

We consider four one-parameter kernels describing a wide range of shapes (Table 1). For computational reasons, when the distance D_{ij} is above a distance threshold Δ the neighbors are considered to have no direct influence on each other and thus their weights are set to 0 (sensitivity to Δ is examined in Text S2.1).

For all kernels, the priors for the parameters of the kernel are identical:

$$\delta \sim \ln\mathcal{N}\left(\mu_\delta, \sigma_\delta^2\right) \quad (2)$$

$$\lambda \sim \ln\mathcal{N}\left(\mu_\lambda, \sigma_\lambda^2\right) \quad (3)$$

with $\mu_\lambda = -2$ and $\sigma_\lambda = 2$ corresponding to a mean λ of 1 (no effect of streets) with a standard deviation on the log scale of 2 and with $\mu_\delta = 1.69$ and $\sigma_\delta = 2$ corresponding to a mean characteristic distance of 40 meters with a standard deviation of 2 on the log scale.

2 The city-block as a spatial unit

According to the marginal distribution of the spatial component of infestation in each household (Eq. 1), the mean of the spatial component of infestation in a household is an arithmetic mean of the mean spatial components of its neighbors. The normalized weights in a row i of the precision matrix can then be considered as additive contributions of the neighbors to the spatial component of the household i. This in turns allows us to determine for each household the percentage of the spatial component explained by neighbors of the same city-block.

We calculate the percent of the spatial component of infestation attributable

to households on the same city block using the following Same Block Index:

$$Same\ Block\ Index = \underset{i}{mean}\left(\frac{\sum\limits_{j\in\Omega_{S_i}} Q_{ij}}{\sum\limits_{j\neq i} Q_{ij}}\right) \quad (4)$$

with Ω_{S_i} the set of households in the same city block as the household i. Most importantly, this measure integrates the "barrier effect" (λ) of the kernel along with a possible "gap effect" which describes the interaction between a sharp shaped kernel and the increased distance to neighbors on different city blocks. Overall, the Same Block Index measures the total impact of streets on the direct spatial correlation of infestation between households.

3 Local component of the infestation predictor

The local component is similar to a classic probit model with a local error term. The indicator matrix C contains as many columns as cofactors and as many lines as households. For each cofactor k present in the house i, $C_{ik} = 1$; $C_{ik} = 0$ elsewhere. The coefficients c_k of each cofactor k is estimated using a normal prior centered at 0 and of variance 100 (precision $\tau_c = 0.01$). A local error term e_i is added for each house i. Its prior is a centered normal distribution of precision τ_v. This precision, common to all households, itself follows a weakly informative gamma prior of scale parameter 1000 and shape parameter 0.001 like the spatial precision.

4 Observation by imperfect inspectors

Each participating household has been examined for insect infestation by one inspector. The sensitivity, q_k, of each inspector is estimated separately from a flat beta prior common to all inspectors: $\alpha_q = 1$ and $\beta_q = 1$ (sensitivity to the prior is examined in Text S2.4). The matrix K contains as many columns as inspectors and as many lines as houses inspected. If the household i is inspected by the inspector k then $K_{ik} = 1$ elsewhere $K_{ik} = 0$. The vector $s = K \cdot q$ then contain the sensitivity of the inspection in each house. A house i is then observed as infested ($z_i = 1$) or not ($z_i = 0$) according to the latent true infestation status y_i and the sensitivity of the inspection in this house s_i.

5 Sampling of the Gaussian Field Latent Class model

We fit our model using a Monte Carlo Markov Chains (MCMC). To estimate the coefficients of the cofactors and the parameters of the precision matrix Q, we use a Metropolis-Hasting sampler. For all other parameters, a conditional distribution can be defined, and a classical Gibb's sampler is used.

Each MCMC is run until the Geweke diagnostic [3] and the Raftery and Lewis diagnostic [4, 5] are satisfied. Estimates are obtained using the second half of each chain. All chains are thinned by a factor 20. For the calculation of the DIC we use only the last thousand iterations of each thinned chain.

References

[1] Rue H, Tjelmeland H (2002) Fitting gaussian markov random fields to gaussian fields. Scandinavian Journal of Statistics 29: 31–49.

[2] Scutari M, Strimmer K (2010) Introduction to graphical modelling. Handbook of Statistical Systems Biology : 235–254.

[3] Geweke J (1992) Bayesian Statistics 4, Clarendon Press, Oxford, UK, chapter Evaluating the Accuracy of Sampling-Based Approaches to the Calculation of Posterior Moments.

[4] Raftery AE, Lewis S (1992) How many iterations in the gibbs sampler. Bayesian statistics 4: 763–773.

[5] Raftery AE, Lewis SM (1996) Implementing mcmc. Markov chain Monte Carlo in practice : 115-130.
