# Supplementary material for: The Effects of City Streets on an Urban Disease Vector
Source: PLoS Comput Biol. 2013 Jan 17;9(1):e1002801. doi: 10.1371/journal.pcbi.1002801 (PMC3547802; doi:10.1371/journal.pcbi.1002801)
Supplement: Text S2 — Sensitivity analyses. We further investigate the sensitivity of the Latent Class Gaussian Field model to the distance threshold , the handling of missing data, the included cofactors, and the inspector sensitivity prior. (PDF) [file pcbi.1002801.s004.pdf]

Supplementary Materials Text S2: Effect of City Streets on an Urban Vector Sensitivity analyses

C.M.Barbu et al.

Contents

1 Sensitivity to distance threshold Δ 1
2 Sensitivity to handling of missing data 3
3 Sensitivity to cofactors 3
4 Sensitivity to inspector sensitivity prior 4

1 Sensitivity to distance threshold Δ

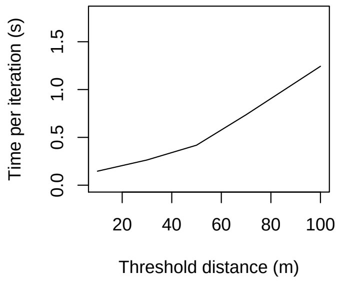

Figure S2.1: Computation time function of the threshold distance
The time for a single iteration is calculated at convergence by dividing the elapsed time of the parameter fitting Markov-chain by the number of iterations.

In the manuscript we report the estimated values of the parameters using a threshold distance of 100m. Here we investigate the sensitivity to this threshold in the range 10-100m using the best fitting model: the one using the exponential spatial kernel. The time per iteration increases exponentially with the threshold distance (Fig. S2.1).

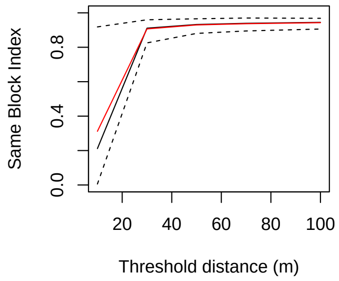

Figure S2.2: Same block index estimate function of the distance threshold
Black solid line: median. Red line: mean. Dashed line: 0.025 and 0975 quantiles. Thresholds at 10, 30, 50, 70 and 100m are tested using the exponential spatial kernel.

The Same Block Index (Fig. S2.2) and the estimates of the kernel parameters are robust to the variations of threshold distance between 50 and 100m (Fig. S2.3).

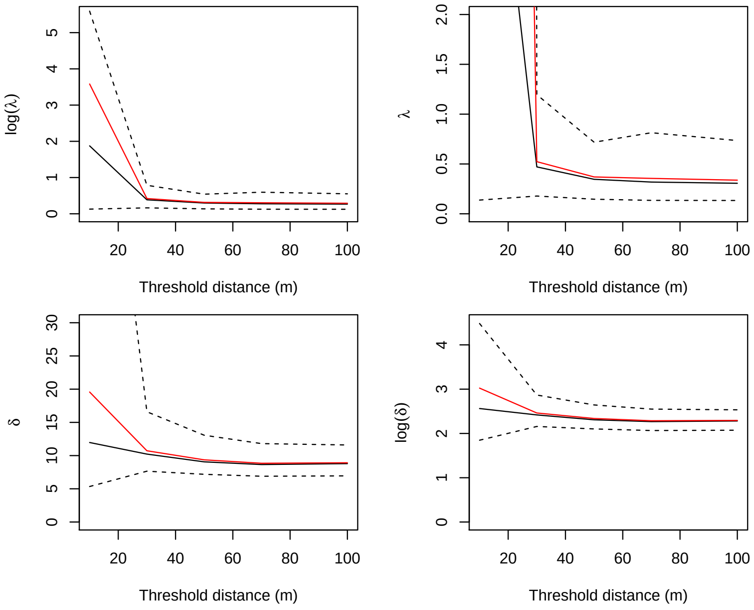

Figure S2.3: Kernel parameters estimates function of the distance threshold
Black solid line: median. Red line: mean. Dashed line: 0.025 and 0.975 quantiles.

2 Sensitivity to handling of missing data

In the manuscript we explicitly consider non-inspected houses for which we have geographical coordinates. In a non-inspected house i, the same model is applied, but the sensitivity of the detection s_i is set to 0. This implies that the probability of infestation is extrapolated by the model using the spatial autocorrelation of the infestation. This allows us to preserve the local density of habitat or texture of the map despite missing information on the infestation and local cofactors.

As this choice potentially affect the evaluation of streets' effect, we here compare estimates provided by alternative models to the ones presented in the manuscript (Tab. S2.1).

Parameter	Reference	Ignored	Considered infested	Considered non-infested
Shape factor, δ	9.3 (7.2 - 12.5)	10.1 (7.6 - 14.0)	15.3 (8.97 - 37.5)	42.3 (10.8 - 229.1)
Streets factor, λ	0.36 (0.15 - 0.72)	0.36 (0.15 - 0.74)	0.17 (0.05 - 0.38)	0.06 (9.5×10^{-2} - 0.16)
Same block index	93.0 % (88.0 - 96.5)	92.5 % (86.7 - 96.5)	95.1 % (90.8 - 97.9)	97.7 % (95.0 - 99.3)

Table S2.1: Estimated autocorrelation for different treatments of missing data For each model the exponential kernel is used with a threshold distance of 50m (see 1). Reference: model used in the main manuscript, the sensitivity of the detection s_i in a non-inspected house i is set to 0. Ignored: non-inspected houses are simply removed from the dataset. Considered infested: $z_i = 1$ in non-inspected houses. Considered non-infested: $z_i = 0$ in non-inspected houses. Estimates are provided with their 95 % CrI.

We consider the simple removal of non-infested houses from the dataset ("Ignored"). As non-participating houses may correspond to a different probability of infestation we also check two extreme cases: all non-inspected houses are infested ($z_i = 1$) or non-infested ($z_i = 0$).

We found that in all cases the estimates of the effect of streets are similar or stronger than the ones presented in the main manuscript. Interestingly the extreme cases where none or all of the non-inspected houses are infested increase the barrier effect of streets significantly (lower λ, higher Same Block Index). This likely indicates that non-inspected houses are also clustered by city-block.

3 Sensitivity to cofactors

To assess the possibility that unmeasured cofactors strongly clustered within blocks confound the effect of streets, we evaluate the sensitivity of the estimated streets effect to known and potential cofactors. First we compare the estimates presented in the manuscript with the estimates obtained without cofactors. Second we introduce a variable clustered by city-block: a binary indicator of large

city-blocks i.e. with more households than the median number per city-block (Tab. S2.2). Adding the five known cofactors decreases the "barrier effect" of streets, but the global effect of streets as reflected by the Same Block Index is only marginally diminished. Finally, the addition of the "Block Size" has a limited additional impact on the estimated effect of streets.

Parameter	Without cofactors	Reference	Reference + Block Size
Shape factor, δ	10.4<br>(8.0 - 13.8)	9.3<br>(7.2 - 12.5)	9.1<br>(7.3 - 11.6)
Streets factor, λ	0.20<br>(0.08 - 0.42)	0.36<br>(0.15 - 0.72)	0.36<br>(0.15 - 0.72)
Same block index	95.2 %<br>(90.8 - 97.8)	93.0 %<br>(88.0 - 96.5)	92.5 %<br>(87.5 - 96.2)

Table S2.2: Impact of cofactors on estimated kernel parameters and same block index

Without cofactors: The analysis is run without cofactors but still maintains the local error term. Reference + Block Size: as our analysis is tailored to binary cofactors we use an indicator variable "big block" for city-blocks containing more than the median number of household per city-block (16 households).

4 Sensitivity to inspector sensitivity prior

In the main text we present results for a flat prior on inspector sensitivity. To assess the robustness of the model to this prior we compare the estimates with a flat prior to those from priors strongly skewed toward low or high detection rates.

These changes in the prior induce modifications of the estimated quality of the inspectors, however, they do not decrease the estimated effect of streets (Tab. S2.3). The strong effect of streets on the autocorrelation of the infestation is robust to the prior on inspector sensitivity.

Estimate	Low	Flat	High
Shape factor, δ	35.0 (12.1 - 155.0)	9.3 (7.2 - 12.5)	8.8 (7.0 - 11.5)
Streets factor, λ	0.0285 *** (6.47×10^{-3} - 6.75×10^{-2})	0.36 ** (0.15 - 0.72)	0.34 ** (0.13 - 0.73)
Same block index	97.9% (96.0 - 99.1)	93.0% (88.0 - 96.5)	93.4% (88.7 - 96.8)
Mean sensitivity, $\bar{q}$	30.0% (27.9 - 32.9)	69.5% (63.0 - 75.6)	89.1% (86.6 - 91.4)

Table S2.3: Impact of inspector sensitivity prior on estimated kernel parameters, same block index and estimated inspector sensitivity

Low: prior $Beta(18, 2)$ on inspectors' sensitivity. Flat: prior $Beta(1, 1)$ on inspectors' sensitivity. High: prior $Beta(2, 18)$ on inspectors' sensitivity. The exponential kernel is used with a threshold distance of 50m (see 1).
